# Cancer and Heart Failure: Dangerous Liaisons

**DOI:** 10.3390/jcdd11090263

**Published:** 2024-08-27

**Authors:** Davide Bertolini, Carmine Pizzi, Erwan Donal, Elena Galli

**Affiliations:** 1 Cardiology Unit, Cardiac Thoracic and Vascular Department, IRCCS Azienda Ospedaliera-Universitaria di Bologna, 40138 Bologna, Italycarmine.pizzi@unibo.it (C.P.); 2 Department of Medical and Surgical Sciences-DIMEC, Alma Mater Studiorum, University of Bologna, 40138 Bologna, Italy; 3 Univ Rennes, CHU Rennes, Inserm, LTSI–UMR 1099, F-35000 Rennes, France; erwan.donal@chu-rennes.fr

**Keywords:** cancer, heart failure, cardiotoxicity, cardiovascular risk factors

## Abstract

Cancer and heart failure (HF) are increasingly relevant worldwide, both from an epidemiologic and clinical point of view. This review aims to explore the relationship between cancer and HF by underscoring risk factors and disclosing the cardiotoxic effects of the current chemotherapy agents. We also deal with the current evidence on the diagnosis and management of HF related to cancer therapy. Finally, we will address the main gaps in knowledge and future perspectives in this field.

## 1. Introduction

Cardiovascular disease (CVD) and cancer are the two most common causes of death worldwide [1]. Among CVD, heart failure (HF) plays a major role in both morbidity and mortality, with a prevalence of roughly 64 million people affected globally [2].

At the same time, cancer is growing as a leading cause of death because of ageing and the spreading of cancer risk factors in the general population. Ninety-three million new cancer cases were diagnosed in 2020, and 28.4 million cases of cancer are expected in 2040—a 47% rise from 2020—with a larger increase in transitioning (64% to 95%) versus transitioned (32% to 56%) countries [3].

HF and cancer share several risk factors and a very complex relationship. On one hand, cancer survivors develop HF because of exposure to cardiotoxic drugs and/or radiotherapy. On the other hand, it has been recently observed that patients with HF have a higher incidence of cancer, the so-called “reverse cardio-oncology” [4].

Given the epidemiologic relevance of HF and cancer and their intertwined causal links, it is easy to see how impactful a clarification of their relationship would be. The evolution of this branch of cardio-oncology requires a tight collaboration between cardiologists and oncologists, both at the bench and bedside levels.

## 2. Cardiovascular Risk Factors and Cancer Development: The “Reverse Cardio-Oncology”

While it could sound rational to assume that patients with HF will die from HF itself or another CVD, many of them will not. Three large randomized clinical trials (RCTs) and some registries have shown that cancer is the most common cause of non-cardiovascular death in HF patients [5,6,7,8,9,10,11,12].

Moreover, HF patients seem to be more prone to develop cancer than the general population without HF. The first evidence of this link comes from the Olmsted Country database. The prospective analysis (1979–2002) of 961 HF patients and 961 sex- and age-matched controls found that HF was associated with a 68% higher risk of developing cancer (HR: 1.68; 95% CI: 1.13 to 2.50), even after adjusting for body mass index (BMI), smoking, and the Charlson co-morbidity index.

Two recent European cohort studies [13,14] including more than 200.000 HF patients and matched controls confirmed the association between HF and cancer, with an incidence rate ranging from 21 to 26% in HF vs. 12 to 16% in controls.

These results have been corroborated by two meta-analyses [15,16], which had the merit to underscore the caveats and traps of previous epidemiological studies on this topic, by showing the potential influence of geographical distribution, ethnicity, age, gender, cardiovascular risk factor prevalence, and follow-up duration on data interpretation. When taking into account all these limitations through the application of subgroup analysis and sensitivity analysis, the relationship between HF and cancer was confirmed, particularly regarding specific cancer subtypes such as breast (HR 1.28, 95% CI: 1.09–1.50), lung (HR, 1.89, 95% CI: 1.25–2.85), hematological (HR 1.63, 95% CI: 1.15–2.33), and colorectal (HR, 1.32, 95% CI: 1.11–1.57) malignancies [15].

The association of HF and cancer seems also to portend higher cancer-related [13,14] and all-cause mortality [17].

The concept that HF can trigger cancer development is intriguing. From a pathophysiological point of view, these links can be attributed to several mechanisms.

First, the chronic inflammation typical of HF can favor carcinogenesis [17,18,19]. Second, oxidative stress, renin–angiotensin–aldosterone system (RAAS) activation, and immune system dysfunction can contribute to the development of both HF and neoplasia [19]. Third, HF and cancer share a common genetic background, depending on both somatic and germline mutations [20,21]. Fourth, HF might be a pro-oncogenic condition by itself, because neurohormonal activation has been related to cancer initiation, progression, and dissemination [18,22].

Finally, HF and cancer share several risk factors, with hypertension, obesity, diabetes, and smoking [19,21,23,24,25,26] being the most important for the development of both cancer and HF.

Previous observations support the inclusion of cancer among the endpoint of HF studies and underscore the importance of lifestyle interventions to reduce the epidemiological burden of HF and cancer in the general population [27].

**Figure 1:**
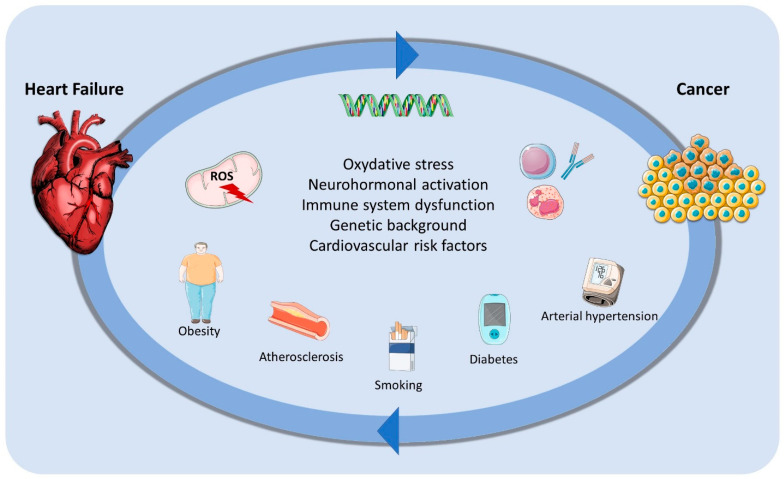
Mutual relationship between heart failure and cancer cardiovascular risk factors. ROS, radical oxygen species.

Concerning this latter point, some real-life studies demonstrated the impact of exercise activity and cardiovascular risk prevention on cancer prevalence [28,29]. In particular, Rasmussen-Torvik L et al. showed that the association of at least six ideal health metrics portended a 51% cancer risk reduction, whereas practicing regular physical activity correlated with a 40% reduction in cancer incidence [29]. Figure 1 summarizes the main mechanisms of the relationship between cancer and HF.

## 3. Heart Failure as a Complication of Cancer Therapy

The etiopathogenesis of HF in cancer patients is often complex, as it depends on several factors which include anticancer regimens, cardiovascular risk factors, the presence of previous CVD, and possibly a shared biology that primes both malignancy and CVD development [18].

The International Cardio-Oncology Society (IC-OS) has recently endorsed a univocal definition for the main forms of cancer therapy-related CV toxicity (CTR-CVT) which encompasses cardiac dysfunction, myocarditis, vascular toxicity, arrhythmias, and hypertension [30]. The same document also provides a classification of the different forms of cancer therapy-related cardiac dysfunction (CTR-CD), included in the 2022 European Society of Cardiology (ESC) society guidelines on cardio-oncology [31].

The current classification of CTR-CD is reported in Table 1.

The following paragraphs will deal with the main classes of chemotherapy agents associated with an increased risk of HF development. As underscored before, it is important to remember that every treatment has its peculiar cardiotoxicity profile. This concerns the onset timing of HF (from days to years after the exposure), mechanism, reversibility, and cardioprotective approaches [32]. The outcome also depends on the specific clinical profile of every patient, underscoring the importance of a patient-tailored approach.

## 4. Mechanisms of Cardiotoxicity of the Main Chemotherapy Agents and Their Association with Heart Failure

### 4.1. Anthracyclines

Anthracyclines are anticancer drugs that are widely used in solid and hematologic malignancies. Their administration is associated with a high percentage of CTR-CVT, with an incidence between 3% and 48%, depending on the specific drug and dose [32].

In cardiomyocytes, anthracycline-induced cardiotoxicity is due to the inhibition of the topoisomerase 2β, which causes the accumulation of double-stranded DNA breaks and mitochondrial dysfunction leading to the accumulation of radical oxygen species and myocyte apoptosis [32].

A large body of evidence has demonstrated that the use of anthracyclines is a strong risk factor for HF [33,34,35,36,37], with a dose-dependent effect [34,36,38]. In a group of 630 patients receiving doxorubicin, the occurrence of HF increased significantly when the cumulative doxorubicin dose exceeded 400 mg/m^2^, going from 26% in patients receiving 550 mg/m^2^ of doxorubicin to 48% in those receiving 700 mg/m^2^ [39,40].

Anthracycline-related CTR-CD can present both symptomatically or asymptomatically [32]. In a meta-analysis, after a median follow-up of 9 years, 6% of patients treated with anthracyclines manifested clinically overt HF, while 18% had subclinical cardiac dysfunction [37]. Interestingly, the development of cardiac dysfunction can occur anytime between the first dose to several years after the treatment [33,35,38,41,42,43]. In a recent study on 2625 patients receiving anthracyclines, 98% of cardiotoxicity events (defined as left ventricular ejection fraction [LVEF] decrease > 10% and/or LVEF <50%) occurred the first year after treatment and were associated with a total recovery of LV function in only 9% of patients [38].

An increase in biomarkers during chemotherapy, in particular of troponin, is an independent predictor of left ventricular (LV) dysfunction at follow-up, providing a rationale for targeted preventive strategies [44].

Age <5 or >65 years when receiving treatment, concomitant chest irradiation, hypertension, diabetes mellitus, smoking, hypercholesterolemia, and obesity are associated with a higher risk of cardiotoxicity and HF [32,45,46].

### 4.2. HER2 Inhibitors

Overexpression of human epidermal growth factor receptor 2 (HER2) in breast cancers and some other solid cancers such as gastric adenocarcinomas is associated with aggressive disease [31].

The specific inhibition of HER2 receptors through either antibodies (e.g., trastuzumab, pertuzumab, and trastuzumab-emtansine) or tyrosin-kinase inhibitors (TKI) (e.g., lapatinib and neratinib) significantly improves prognosis in patients with HER2 mutations [47,48].

A study pooling three trials on trastuzumab showed that 8.7% of patients developed asymptomatic or mildly symptomatic (NYHA class 2) HF, and 2.3% of patients developed severe (NYHA classes 3 and 4) HF [49]. In a meta-analysis of eight trials on patients receiving trastuzumab, 2.5% (RR 5.11, 90% CI 3.00–8.72) developed HF, and 11.2% (RR 1.83, 90% CI 1.36–2.47) presented LV dysfunction, respectively [48].

Anti-HER-2-related cardiotoxicity has been classically thought to be dose independent [50]. However, some studies show that longer treatment is associated with a double rate of LV dysfunction [51,52].

HER-2-related cardiotoxicity typically occurs during treatment, with very low rates of late-onset HF in patients with low cardiovascular risk, and mostly resolves after treatment discontinuation [48,51,52,53,54].

Exposure of cardiomyocytes to clinically relevant doses of trastuzumab revealed mitochondrial dysfunction and impaired contractile function, with no cardiomyocyte death, unraveling the reversibility of the cardiotoxic effects [55]. The deletion of ErbB2 (HER2 gene) increases cardiac susceptibility to anthracyclines cardiotoxicity [56,57], which explains why previous or concomitant exposure to anthracyclines is a known risk factor for anti-HER2-related CTR-CVD. In a retrospective study, patients treated with anthracyclines and anti-HER2 had an incidence of a composite of cardiac dysfunction and HF of 6.2% at 1 year and 20.1% at 5 years [35]. A short delay between anthracyclines and anti-HER 2, arterial hypertension, low baseline LVEF, and older age represent risk factors for developing HF after anti-HER2 agents [50,58].

### 4.3. VEGF Inhibitors

Vascular endothelial growth factor inhibitors (VEGFi) are used for the treatment of solid cancers and include monoclonal antibodies (e.g., bevacizumab) as well as TKI (e.g., sunitinib and sorafenib) with different specificities for VEGF receptors.

The more prominent cardiotoxic effect of VEGFi is arterial hypertension [59,60], nevertheless most of these drugs can induce cardiac dysfunction. In a trial of 2591 patients with triple-negative breast cancer, the administration of bevacizumab induced LV dysfunction in 2% of patients and HF (NYHA class 3 or 4) in 1% of patients [61]. A meta-analysis of five studies incuding 3784 patientsshowed that high-grade HF occurred in 1.6% (RR = 4.74, 95% CI: 1.66–11.18) of patients receiving bevacizumab, independently of the administered dose [62].

The main evidence on the cardiotoxicity of VEGF-TKIs concerns sunitinib. Sunitinib administration is associated with a 9 to 19% risk of developing LV dysfunction [63,64]. A meta-analysis of 6935 patients treated with sunitinib showed an incidence of 4.1% and 1.5% of all- and high-grade HF, respectively [32,65]. However, a retrospective study found that nearly half of sunitinib-related LV dysfunction cases recovered within 5 percentage points of baseline during the follow-up [66].

A meta-analysis including several anti-VEGF drugs (axitinib, cediranib, pazopanib, ramucirumab sorafenib, sunitinib, and vandetanib), including 10553 patients from 36 clinical trials showed an incidence of all- and high-grade HF of 3.2% (OR = 2.37, 95% CI 1.76–3.20) and 1.4% (OR = 3.51, 95% CI 1.74–7.05), respectively. In this study, pazopanib (6.1%) and cediranib (5.9%) were associated with the higher incidence of HF episodes, while vandetanib (0.4%) and ramucirumab (0.4%) were safer. The risk of HF was not influenced by tumor type [67].

Anti-VEGF drugs act on several signaling pathways, making it difficult to clarify the complex mechanisms of their cardiotoxicity [59,68,69]. Arterial hypertension seems to be a predisposing factor for VEGF inhibitor-induced HF [66]. This led some authors to speculate that at least a part of HF cases in these patients could be reduced with an effective anti-hypertensive regimen [32].

### 4.4. Hematological Therapies

BCR-ABL inhibitors such as imatinib, bosutinib, dasatinib, nilotinib, and ponatinib are small-molecule TKIs used in chronic myeloid leukemia and some gastrointestinal stromal tumors. First-generation BCR-ABL inhibitors are not associated with significant cardiotoxic effects [70,71], while second- and third-generation BCR-ABL inhibitors such as dasatinib can cause group 1 pulmonary hypertension and HF [72,73]. Cardiotoxicity risk is higher in elderly patients (>65 years) and in the case of concomitant cardiovascular risk factors such as diabetes, arterial hypertension, or previous coronary artery disease.

Bruton tyrosine kinase (BTK) inhibitors are used in lymphoid malignancies.

Ibrutinib administration is associated with an increased risk of atrial fibrillation (AF) requiring intervention [74]. HF occurs in nearly 5% of patients receiving ibrutinib [75] and might be attributed to AF-related tachycardia and induced arterial hypertension, but also the direct cardiotoxic effect of the drug [76]. The off-target inhibition of myocardial tyrosine kinases can lead to myocardial cell disarray, fibrosis, disruptions in calcium signaling, and death favoring the development of HF [77]. Acalabrutinib, a second-generation BTK inhibitor with greater BTK selectivity, is also associated with an increased incidence of AF, but with a lower rate of HF episodes [78].

Proteasome inhibitors (PIs), such as bortezomib, carfilzomib, and ixazomib, are a mainstay therapy for patients with newly diagnosed multiple myeloma as well as relapsed disease. Carfilzomib, a second-generation irreversible PI, has a more potent and long-lasting therapeutic effect than bortezomib. The ENDEAVOR trial has shown that patients with multiple myeloma receiving carfilzomib and dexamethasone have a 10.8% risk of developing HF compared to the 4.1% risk of those receiving bortezomib and dexamethasone [79]. These data are confirmed in a large meta-analysis showing that bortezomib (OR 1.18, 95% CI 0.73–1.92) and ixazomib (OR 1.56, 95% CI 0.84–2.90) did not increase the risk for all-grade cardiotoxicity, while carfilzomib (OR 2.68, 95% CI 1.63–4.40) did, even if no specific analysis was made on HF [80].

Cardiomyocytes’ susceptibility to carfilzomib is due to several mechanisms including protein homeostasis disruption, mitochondrial dysfunction, and increased oxidative stress [81]. Elderly patients with cardiovascular risk factors and previous LV dysfunction are at increased risk of PI cardiotoxicity [82]. LV dysfunction may be reversible after carfilzomib discontinuation and HF treatment [83]; however, no consensus exists about the risk related to PI treatment resumption.

### 4.5. BRAF/MEK Inhibitors

B-type rapidly accelerated fibrosarcoma (BRAF) inhibitors (vemurafenib, dabrafeniband, and encorafenib) and mitogen-activated extracellular signal-regulated kinase (MEK) inhibitors (trametinib, cobimetinib, binimetinib, and selumetinib) are used, alone or in combination, in patients with BRAF-mutated melanoma. The incidence of LV systolic dysfunction in clinical trials on these drugs has been reported as 2% to 12% [84]. In a meta-analysis of five RCTs (2317 patients with melanoma), BRAF/MEK inhibitors combined therapy was associated with a higher risk of LV dysfunction, particularly in younger patients (RR 26.50, 95% CI 3.58–196.10) [85]. This side effect is probably due to a microvascular rarefaction/damage mechanism [86].

Pre-existing cardiovascular disease, age, and traditional cardiovascular risk factors such as hypertension, diabetes, and smoking may predispose patients to BRAF/MEK inhibitor cardiotoxicity [87].

### 4.6. EGFR Inhibitors

Epidermal growth factor receptor (EGFR) is a tyrosine kinase often mutated in non-small-cell lung cancer and other solid tumors. EGFR inhibitors include small molecules such as erlotinib, afatinib, gefitinib, osimertinib, and monoclonal antibodies such as cetuximab. HF is rarely associated with first- and second-generation EGFR inhibitors. Osimertinib, a third-generation EGFR inhibitor, carries a higher risk for new-onset HF [88].

A large retrospective study on the FDA adverse events reporting system related a 5.5% (4.2–7.1) odds ratio of heart failure for osimertinib, vs. other EGFR-TKIs, with a median time to onset of LVEF decline of 5.5 months [89].

### 4.7. Immune Checkpoint Inhibitors

Many cancers evade the immune response by acting on specific proteins that regulate T-cell differentiation, such as CTLA-4 (cytotoxic T lymphocyte-associated protein 4), PD-1 (programmed cell death 1), and PD-L1 (PD-1 ligand 1). Immune checkpoint inhibitors (ICI) (ipilumab, pembrolizumab, nivolumab, cemiplimab, atezolizumab, avelumab, and durvalumab) are monoclonal antibodies that target these proteins and induce the activation of cytotoxic T-lymphocytes against cancer cells [90].

The main mechanism of ICI-induced cardiac damage is due to T-cell hyperactivation and infiltration of the myocardium, but indirect mechanisms involving vascular damage, cytokines release, and auto-antibodies are also involved [90].

Despite ICI-related myocarditis often being described as the main cause of immunotherapy-induced LV dysfunction [91,92], a meta-analysis of 63 RCTs found that HF related to ICI had a higher incidence than myocarditis (8.7 vs. 3.2 per 1000 patients) [93]. In a small retrospective study on ICI cardiotoxicities, 79% of patients presented LV dysfunction, whereas a Takotsubo-like appearance occurred in 14% [94]. Interestingly, despite most adverse cardiac events after ICI therapy arising early within a median of 65 days from the first administration, late events can occur [95,96] with LV systolic dysfunction being the most frequent type of late adverse event [95].

Table 2 provides an overview of the main anticancer regimens associated with heart failure.

## 5. Heart Failure Prevention in Cancer Patients

Preventing HF in cancer patients receiving cardiotoxic treatments fundamentally involves three actions, all of which are best carried out in the context of an integrated cardio-oncology approach [97].

The first step is the identification and treatment of cardiovascular risk factors and pre-existing cardiovascular disease [31]. The second step is the assessment of the CV toxicity risk related to chemotherapy. Recent European Society of Cardiology recommendations in cardio-oncology suggest applying the Heart Failure Association (HFA)-International Cardio-Oncology Society (ICOS) tool to determine the pre-treatment risk of CTR-CVT [31]. This approach enables the identification of patients with a high or very high risk of developing cardiotoxicity, who might need cardiology advice before starting anticancer treatment and deserve close follow-up.

The third step concerns the application of specific prevention strategies to limit the occurrence of cardiotoxicity. The large majority of evidence in the literature focuses on the application of neurohormonal therapies (e.g., angiotensin-converting enzyme inhibitors [ACEIs], angiotensin receptor blockers [ARBs], and beta-blockers) [98,99,100] in the prevention of LV dysfunction and HF in patients receiving anthracyclines or anti-HER2 agents.

The heterogeneities of current data reflect the differences in the chemotherapy regimens, baseline risk of cardiotoxicity, and cardioprotective approach among the studied populations.

The final objective is to reduce the burden of HF and cardiac disease in oncologic patients allowing them to receive the best antitumor therapy with the lowest rate of side effects and treatment interruptions.

### 5.1. Angiotensin-Converting Enzyme Inhibitors and Angiotensin Receptor Blockers

Two meta-analyses in patients receiving anthracyclines found that ACEIs have the strongest protective effect on LV function, without reducing the incidence of other cardiotoxic events [101,102].

On the other hand, the multicenter, prospective CARE trial cannot demonstrate the cardioprotective effect of candesartan and carvedilol combined therapy in high-risk patients receiving anthracycline-based chemotherapy [103].

The prospective PRADA trial which randomized patients receiving adjuvant anthracyclines with or without trastuzumab to candesartan, metoprolol succinate, or matching placebos found that candesartan protects against an early decline in global LV function [99]. These results make it difficult to firmly confirm the cardioprotective effects of angiotensin receptor blockers in high-risk patients receiving cardiotoxic chemotherapy.

### 5.2. Beta-Blockers

The cardioprotective effect of beta-blockers in patients receiving anthracyclines is an object of debate. A network meta-analysis showed that spironolactone is more cardioprotective than beta-blockers (mean difference in LVEF = 1.98, 95% CI 0.15–3.81, *p* = 0.03) [104], whereas another large meta-analysis found that carvedilol does not have any effect on LVEF (mean difference in LVEF under carvedilol vs. placebo: 1.74; 95% CI = −0.18–3.66, *p* = 0.08), but it could diminish the incidence of clinically overt cardiotoxicity (OR, 0.42; 95% CI, 0.20–0.89; *p* = 0.02) [105]. Similarly, the CARE trial did not prove the cardioprotective effect of carvedilol in patients receiving anthracyclines [103].

On the other hand, a placebo-controlled trial including 468 women with breast cancer receiving combined treatment with anthracyclines and anti-HER2, lisinopril, or carvedilol exhibited reduced events rate (37% and 31%, respectively, compared to 47% in patients receiving placebo). Patients taking these cardioprotective drugs also experienced fewer interruptions in trastuzumab than those on placebo [100].

Another beta-blocker, bisoprolol, seems to have a cardioprotective effect in patients receiving a regimen combining anthracyclines and anti-HER2 or in patients receiving anti-HER2 alone.

The MANTICORE trial, which featured 77% of patients receiving trastuzumab in an anthracycline-free regimen, is probably the most specific study on the topic and demonstrated that treatment with perindopril (β = 2.59, *p* = 0.016) or bisoprolol (β = 4.56, *p <* 0.001) vs. placebo prevented the reduction in LVEF during follow-up [106].

### 5.3. Sacubitril Valsartan

The benefit of sacubitril/valsartan for HF prevention in patients with a higher risk of CTR-CVT is not fully established.

The prospective randomized MAINSTREAM trial NCT05465031 will clarify if the higher tolerated dose of Sacubitril/Valsartan can prevent cardiotoxicity in patients with breast cancer undergoing anthracyclines+/-anti-HER2 regimens [107].

### 5.4. Sodium–Glucose Co-Transporter Inhibitors

Experimental studies have shown that empagliflozin and dapagliflozin can contrast the cardiotoxic effects of anthracyclines through cholesterol-lowering, anti-inflammatory, and endothelium-stabilizing properties [108,109].

From a clinical point of view, the multicenter randomized PROTECTAA trial (NCT06304857) will provide further evidence on the protective role of dapagliflozin in breast cancer patients undergoing anthracycline-based chemotherapy.

### 5.5. Other Mechanisms of Cardioprotection

Dexrazoxane is a bisdioxopiperazine that decreases the formation of anthracycline–iron complexes and the production of reactive oxygen species which are harmful to the surrounding cardiac tissue [110].

Dexrazoxane has been shown to provide cardioprotection in patients with high cardiotoxicity risk undergoing anthracycline chemotherapy [31,111]. In a meta-analysis of breast cancer patients receiving anthracyclines, with or without trastuzumab, dexrazoxane reduced the risk of HF, even if it did not improve overall survival [112].

### 5.6. Dyslipidemia and Diabetes Treatment

Conflicting data exist about the cardioprotective effect of statins in patients receiving anthracyclines. In the recent STOP-CA randomized clinical trial, atorvastatin treatment was associated with a significantly lower rate of LV dysfunction (OR 2.9; 95% CI, 1.4–6.4) without impact on the incidence of new-onset HF (3% with atorvastatin, 6% with placebo, *p* = 0.26) [113].

Finally, an intriguing retrospective study found that in patients with diabetes and cancer treated with anthracyclines, metformin reduced HF and overall mortality [114].

### 5.7. Practical Approach to Cardioprotection

The largest amounts of data on cardioprotection have been obtained in patients receiving anthracyclines or anti-HER2 therapy. In patients receiving other potential cardiotoxic therapeutic regimens, the evidence of the cardioprotective effects of drugs traditionally used in HF management is weaker.

However, current guidelines endorse the use of ACEIs, ARB, and beta-blockers for the primary prevention of HF in high- and very high-risk patients receiving cardiotoxic targeted cancer therapies (VEGF inhibitors, RAF/MEK inhibitors, PI, dasatinib, ponatinib, and osimertinib) with a class IIA level of evidence C. In the same context, statins have class IIA level of evidence C [31].

Table 3 summarizes the main cited clinical trials focusing on HF prevention in cancer patients (review and meta-analysis are excluded from this table).

## 6. Heart Failure Diagnosis in Cancer Patients

The timely diagnosis of HF in patients receiving oncological treatment is important because it allows for early therapeutic interventions, prevents advanced disease, and minimizes the risk of cancer therapy interruption [115,116].

This goal can be achieved through the careful follow-up of high-risk patients, the screening for typical HF signs and symptoms, and the periodic evaluation of LV function.

In many patients, CTR-CVT is not associated with overt HF symptoms but manifests through asymptomatic impairment of LV performance.

Because of its large availability, cost-effectiveness, and reliability, transthoracic echocardiography (TTE) is the reference method for the assessment of LV function in cancer patients [117]. Three-dimensional assessment of LV size and function at TTE should be privileged when available in experienced centers for the follow-up of patients [118]. Cardiac magnetic resonance has demonstrated a prognostic value and should be considered to assess LV function when TTE is not diagnostic [119,120], whereas isotopic ventriculography should be avoided [31].

Depressed LVEF and increased indexed LV end-diastolic volume at baseline are known to be associated with a higher risk of HF in patients treated with trastuzumab [51,53,87] or anthracyclines [121].

Compared to LVEF, global longitudinal strain (GLS) has shown to be a more sensitive predictor of cardiotoxicity in patients receiving anthracyclines [122,123]. A meta-analysis of 21 studies on different types of cancer treated with anthracyclines with or without trastuzumab demonstrated a good prognostic value of both absolute and relative reduction in GLS for CTRCD [124], supporting the application of GLS for the routine monitoring of chemotherapy-induced LV dysfunction.

However, the 3-year results of the SUCCOUR trial failed to demonstrate the advantage of GLS-guided vs. LVEF-guided cardioprotective treatment on LVEF variation in patients undergoing anthracycline chemotherapy [125], also questioning the risk of an excessive GLS-guided interruption of chemotherapy [72].

Biomarkers such as BNP, NT-pro-BNP, and cTnT/I can be useful in evaluating baseline risk and monitoring the cardiotoxic effects of chemotherapy. Even if an isolated rise of biomarkers is rarely sufficient to discontinue cancer therapy, it can contribute to the multidisciplinary discussion on follow-up timing, cardioprotective treatment, and possibly orienting towards second-line cancer treatments [126]. Of note, in a large meta-analysis, both BNP/NT-pro-BNP and troponins increased after cancer therapy, but only troponins were associated with a higher risk of overt LV dysfunction [127].

While there is consensus on the utility of baseline measurement of biomarkers [87], debate exists on the best timing and frequency during therapy [126], such as on the value of biomarkers in guiding the initiation of cardioprotection therapies.

The small, multicenter, open-label CARE trial evidenced that in patients receiving anthracycline-based chemotherapy presenting an increase in troponin I during therapy, the association of candesartan and carvedilol did not prevented LV dysfunction (6-month difference in LVEF between groups: −0.37%, 95% CI: −3.59–2.85%, *p* = 0.82) [103].

**Figure 2:**
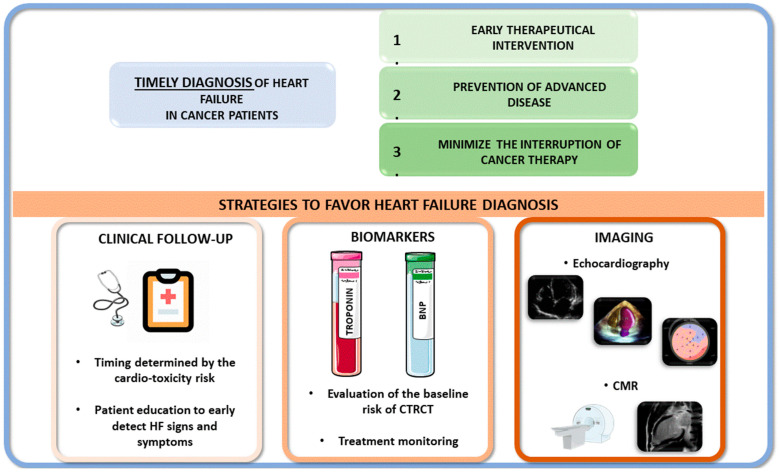
**Upper panel**: advantages of a timely diagnosis of heart failure (HF) in cancer patients. **Lower panels**: main strategies to favor HF identification in cancer patients. **Left**: regular clinical follow-up should be scheduled according to cardiotoxicity risk and patient education to detect symptoms and signs of HF. **Center**: biomarkers are useful before cancer treatment initiation to establish the risk of cardiotoxicity and during cancer treatment for treatment monitoring. **Right**: echocardiography and CMR have a pivotal role in evaluating the evolution of left ventricular function and detecting asymptomatic cardiotoxicity. CMR, cardiac magnetic resonance; CTRCT, chemotherapy-related cardiac toxicity.

Figure 2 summarizes the advantages of a timely diagnosis of HF in cancer patients (upper panel) and the main strategies to favor the identification of HF (lower panel).

## 7. Management of Chemotherapy-Induced Heart Failure

It seems reasonable that HF following cardiotoxic drugs should be managed according to HF general principles [42,128]. In a real-life study conducted on 128 patients who developed HF under different chemotherapy regimens, guideline-based up-titration of HF medical therapy was associated with a 94% improvement in LVEF and NYHA class [129].

Current data focus on the use of ACE-Is, ARBs, beta-blockers, and aldosterone antagonists in chemotherapy-induced HF. The benefit of sacubitril/valsartan or the sodium-glucose co-transporter (SGLT-2) inhibitors in this field is not fully established.

A small Spanish retrospective multicentric registry has shown that sacubitril/valsartan treatment is safe in patients developing CTR-CD and is associated with significant improvement in N-terminal pro-B-type natriuretic peptide levels (1552 [692; 3624] vs. 776 [339; 1458] pg/mL, *p <* 0.01), NYHA class (2.2 ± 0.6 vs. 1.6 ± 0.6, *p <* 0.01), and LVEF (33 [27; 37] vs. 42 [35; 50]%, *p <* 0.01) [130].

Finally, in the case of chemotherapy-induced HF, it is mandatory to discuss the discontinuation of chemotherapy. Current data in this field rely essentially on the consensus of experts. In the case of anthracyclines-induced cardiotoxicity, the overall approach is restrictive, leading to chemotherapy interruption in moderate-to-severe cardiotoxicity. In the case of mild symptomatic cardiotoxicity or HF recovery under cardioprotective therapy, the continuation/resumption of chemotherapy should be discussed by a multidisciplinary team [31].

Anti-HER2-induced moderate-to-severe cardiotoxicity requires temporary discontinuation of chemotherapy, with resumption when LV function recovers [31]. Several studies have shown that LV function improvement is frequent in patients receiving trastuzumab [131,132], which can explain why anti-HER2 chemotherapy should be continued in the case of LVEF> 40% [31].

### Device Therapy and Advanced HF Therapy in Cancer Patients

Because of the improvement in cancer and HF therapy, the number of cancer survivors continues to grow globally, and some of these patients might present persisting HF symptoms despite optimized medical therapy.

Cardiac resynchronization therapy (CRT) has a class I indication in patients with HF, LVEF < 35% and a QRS duration > 130 ms. In the specific subset of patients with chemotherapy-related HF, the small MADIT-CHIC trial has shown that CRT was associated with a significant improvement in LVEF and size at 6-month follow-up [133].

Cancer patients are underrepresented in intracardiac defibrillator (ICD) trials. A Danish large population cohort study demonstrated that the rate of ICD-appropriate therapy is similar independently of the cancer history of patients when an ICD is implanted in primary prevention. In the case of a secondary prevention ICD, the rate of ICD-appropriate therapy is significantly higher in cancer patients, with almost 60% of patients receiving at least an ICD-shock during follow-up [134].

In cancer patients and survivors, the presence of challenging vascular access and the higher risk of infectious complications might favor the implantation of a subcutaneous ICD over a traditional ICD [135].

However, CRT and/or the implantation of an ICD is discouraged in patients with less than 1-year life expectancy.

Concerning advanced HF therapy, the large multicenter INTEMACS registry has shown that patients with advanced HF due to chemotherapy are more often women, with a higher prevalence of biventricular dysfunction. In these patients, mechanical cardiac support is often a destination therapy and the survival after implantation is similar to that observed in other HF etiologies [136].

Heart transplantation is the definitive therapy for advanced end-stage HF. An arbitrary 5-years of cancer-freedom is commonly required to allow transplantation. This requirement, together with the evidence of concerns for malignancy recurrence in the setting of immunosuppression makes patients with chemotherapy-related advanced HF less often eligible for heart transplant [137]. Retrospective data from transplant registries show that patients with chemotherapy-induced advanced HF undergoing heart transplant had a lower risk of rejection and complications such as kidney failure but an increased risk of infections [138,139]. Cancer recurrence is a rare cause of death, whereas restrictive cardiomyopathy due to previous mediastinal irradiation is associated with worse survival [140].

## 8. Follow-Up after Cancer

The occurrence of HF in cancer survivors is difficult to predict. However, several cohort studies have shown that patients who experienced cancer during childhood have a higher risk of congestive HF (RR 15.1, 95% CI 4.8–47.9) [141] and HF death (RR 18.2, 95% CI 3.9–84.2) [142] compared to the age-matched population. In a study on 1362 childhood cancer survivors with a median follow-up time of 17 years, 126 of them (9.3%) were diagnosed with some form of cardiomyopathy [143].

Despite the risk of cardiac death being more pronounced when cancer is diagnosed at a younger age, adult cancer survivors are also prone to experience CV events [144].

ESC guidelines on cardio-oncology endorse a follow-up based on the risk stratification of HF based on the initial cancer therapeutic regimen [31]. Moderate-risk childhood and adolescent cancer survivors are advised to undergo a cardiovascular review with echocardiography every 5 years [31], whereas for high-risk patients its every 2 years, with lifelong surveillance [145]. In high-risk adult cancer survivors, cardiology follow-up with echocardiography should be scheduled 1, 3, and 5 years after discontinuing cancer therapy, and then every 5 years.

Incorporating LVEF in the evaluation seems to improve the prognostic power for LV systolic dysfunction at a 10-year follow-up [146].

As of now, there are not specific management strategies for HF occurring late after chemotherapy exposure.

In adult survivors with mild-or-moderate CTRCD, a suspension of HF drugs can be attempted, provided that LVEF has fully recovered and serum biomarkers are within the normal range. Conversely, failure to recover, as well as those with severe and very severe CTRCD, should continue long-term treatment [31]. Since both pre-cancer and post-cancer cardiorespiratory fitness impairment is a strong independent predictor of future all-cause death and cardiovascular death [147,148], exercise therapy (ET) has gained interest over the years as a strategy to improve prognosis in cancer survivors. A meta-analysis of 48 RCTs showed that exercise is associated with a significant improvement in cardiorespiratory fitness [149]. High-intensity training is well tolerated but does not produce a higher peak VO2 in comparison to moderate-intensity training [150]. Considering clinical endpoints, ET in a post-adjuvant setting demonstrated not only a reduction in all-cause mortality but also in cardiovascular events in different types of cancers [151,152]. Data about ET’s effects on the prevention of late HF are lacking. However, it must be noted that HFpEF has a higher incidence than HFrEF in certain populations of cancer survivors, like postmenopausal women with breast cancer [153]. In these patients, ET could act on modifiable factors of HFpEF such as excessive body weight and hypertension.

## 9. Future Perspectives

The 2023 Global Cardio-Oncology Summit identified the prediction of CTR-CVD and the identification of mechanisms linking cardiovascular disease and cancer as main research priorities in the field [154].

There are several potential ways to accomplish these objectives.

(1) Planning studies including patients with both HF and cancer, two conditions that are mutually exclusive in previous clinical trials [155,156];
(2) Recognizing the role of genetics and hereditable tracts in identifying patients at risk of developing HF during chemotherapy and discovering new therapeutic targets [157];
(3) Disclosing the role of cardiovascular drugs in the prevention of HF in cancer patients but also their possible role in cancer prevention and outcome [155,158];
(4) Adopting rigorous translational research protocols, trying to consider the heterogeneity observed in real-life patients [154];
(5) Underscoring the impact of psychosocial stress, gender, ethnicity, and social status on the relationship between HF and cancer.

## 10. Conclusions

Cancer and HF are increasingly relevant worldwide, both from an epidemiologic and clinical point of view. The dramatic advancements in cardio-oncology in the last years allowed us to disclose the tight relationship existing between cancer and HF.

However, until now, the management of cancer patients developing HF and, vice-versa, of HF patients diagnosed with cancer relies on the opinion of experts more than on randomized clinical trials. Studies in this field have often been flawed by limitations, such as the enrollment of unselected patient populations and the inconsistent definitions of cardiac dysfunction and HF.

The efficient implementation of cardio-oncology in the upcoming years will rely on the alliance between bench and bedside research and the strict collaboration between cardiologists and oncologists. This will favor the adoption of a patient-tailored comprehensive approach and allow the disclosure of effective and specific treatments in the field.

## Figures and Tables

**Table 1:** Definition of cardiotoxicity according to the Heart Failure Association (HFA) of the ESC in collaboration with the International Cardio-Oncology Society (ICOS).

Symptomatic		
	**Very severe**	HF requiring inotropic support, mechanical circulatory support, or transplantation
	**Severe**	HF hospitalization
	**Moderate**	Need for outpatient intensification of diuretic and HF therapy
	**Mild**	Mild HF symptoms, no intensification of therapy required
**Asymptomatic**		
	**Severe**	New LVEF reduction to >40%
	**Moderate**	New LVEF reduction by ≥10 percentage points to an LVEF of 40–49% OR New LVEF reduction by <10 percentage points to an LVEF of 40–49% AND either new relative decline in GLS by >15% from baseline OR new rise in cardiac biomarkers
	**Mild**	LVEF ≥ 50% AND new relative decline in GLS by >15% from baseline AND/OR new rise in cardiac biomarkers

GLS, global longitudinal strain; LVEF, left ventricular ejection fraction; HF, heart failure.

**Table 2:** Overview of the main anticancer regimens associated with heart failure.

Drug Family	Type of Treated Cancer	Cardiotoxicity	Main HF Risk Factors	Percentage of Cardiotoxicity	Citations Author, Journal, Year (Ref.)
**Anthracyclines** Doxorubicin Daunorubicin Epirubicin Idarubicin Mitoxantrone	Breast, small-cell lung, bladder, esophagus, stomach, liver and thyroid, leukemias, lymphomas, sarcomas	Cardiomyocytes topoisomerase 2β irreversible inhibition leading to ROS release and myocyte apoptosis. Dose-dependent cardiotoxicity	Previous HF, CTR-CVT, advanced age, previous radiotherapy, previous anthracyclines exposure, LVEF < 50%	3 to 48% HF incidence, depending on the dose, specific drug, and risk profile of patients	Boeker et al. Br. J. Cancer 2018 [33] Boeker et al. Eur. J. Heart Fail. 2020 [34] Bowles et al. Natl. Cancer Inst. 2012 [35] Jacobse et al. Breast Cancer Res. Treat. 2021 [36] Lotrionte et al. Am. J. Cardiol. 2013 [37] Cardinale et al. Circulation 2015 [38] Von Hoff DD. Ann. Intern. Med. 1979 [39] Swain et al. Cancer 2003 [40] Pinder et al. J. Clin. Oncol. 2007 [41] Thavendiranathan et al. J. Clin. Oncol. 2016 [42] J. Clin. Oncol. 2008 [43]
**HER-2 inhibitors** Antibodies: Trastuzumab, Pertuzumab, TrastuzumaB-emtansine TKI-inhibitors Lapatinib Neratinib Tucatinib	Breast cancers Metastatic gastric adenocarcinoma	Inhibition of the human epidermal receptor 2 leading to mitochondrial dysfunction and impaired contractile LV function, without cardiomyocyte death. Dose-independent, often reversible cardiotoxicity	Previous HF, CTR-CVT, advanced age, previous radiotherapy, previous exposure to trastuzumab, LVEF < 50%	8.7% incidence of asymptomatic or mildly symptomatic HF; 2.3% incidence of severe HF	Slamon et al. N. Engl. J. Med. 2001 [47] Moja et al. Cochrane Database Syst. Rev. 2012 [48] De Azambuja Breast Cancer Res. Treat. 2020 [49] De Asambuja etal. J. Clin. Oncol. 2014 [51] Goldhirsch et al. The Lancet 2013 [52] Romond et al. J. Clin. Oncol. 2012 [53] Advani et al. J. Clin. Oncol. 2016 [54] Kitani et al. Circulation 2019 [55]
**VEGF inhibitors** Monoclonal antibodies: Bevacizumab TKI-inhibitors Axitinib Cabozantinib Lenvatinib Pazopanib Regorafenib Sorafenib Sunitinib Vandetanib	Solid cancers, such as renal, thyroid, and hepatocellular carcinomas	Endothelial dysfunction and impaired myocardial perfusion, increased afterload	Previous HF, CTR-CVT, previous anthracyclines exposure, VTE or PE, LVEF < 50%, QRS width > 480 msec, age ≥ 75 years, arterial hypertension	Hypertension is the main CV side effect (class effect). HF is common (1–10%) for bevacizumab, axitinib, levantinib, and sorafenib. HF is uncommon (<1%) with the other drugs	Touyz et al. Npj Precis. Oncol. 2018 [59] Abdel-Qadir Cancer Treat. Rev. 2017 [60] Cameron et al. Lancet Oncol. 2013 [61] Choueiri J. Clin. Oncol. 2011 [62] Mozter et al. N. Engl. J. Med. 2013 [63] Chu et al. The Lancet 2007 [64] Richards et al. J. Clin. Oncol. 2011 [65] Ewer et al. Eur. J. Cancer 2014 [66] Qi et al. Br. J. Clin. Pharmacol. 2014 [67] Maurea et al. J. Cardiovasc. Med. 2016 [68] Force et al. Nat. Rev. Cancer 2007 [69]
**BCR-ABL multi-targeted tyrosine kinase inhibitors** 1st generation: Imatinib 2nd generation: Dasatinib, Bosutininb 3rd generation: Ponatinib	Chronic myeloid leukemia	Off-target TKI leading to SERCA dysfunction, mitochondrial dysfunction, and myocyte death; endothelial dysfunction, pulmonary capillary medial hypertrophy, pulmonary infiltrates, and pulmonary hypertension	Previous HF, CTR-CVT, previous anthracyclines exposure, arterial vascular disease, previous arterial thrombosis under TKI, PH, LVEF < 50%, QRS width > 480 msec, age ≥ 75 years, CVD 10-year risk score 0.20%, current smoking	HF is common (1–10%) with dasatininb and ponatininb; uncommon (<1%) with imatininb. Pulmonary hypertension is common with dasatinib and ponatinib; uncommon with bosutininb	Verweij et al. Eur. J. Cancer 2007 [70] Druker et al. N. Engl. J. Med. 2006 [71] Barber et al. Hematology 2017 [73]
**Bruton tyrosine kinase inhibitors** Ibrutininb Acalabrutininb	Chronic lymphocytic leukemia, B-cell malignancies, Waldenström macroglobulinemia, and marginal zone lymphomas	Off-target myocardial TKI leading to myocardial cell disarray, fibrosis, disruptions in calcium signaling, and death	Previous HF/cardiomyopathy, AF, myocardial fibrosis	Up to 7% with ibrutininb; <1% with acalabrutininb	Sestier et al. Curr. Oncol. Rep. 2021 [74] Salem et al. J. Am. Coll. Cardiol. 2019 [75] Abdel-Qadir et al. J. Clin. Oncol. 2021 [76] Quartermaine et al. JACC CardioOncology 2023 [77] Brown et al. Haematologica 2021 [78]
**Proteasome Inhibitors** Bortezomib Carfilzomib	Multiple myeloma	Myocyte proteasome instability, mitochondrial dysfunction, ROS accumulation, genetic instability, sarcomericdysfunction	Previous HF, CTR-CVT, previous anthracyclines exposure, arterial vascular disease, VTE or PE, previous cardiac toxicity under PI or immunomodulators, PH, LVEF < 50%, cardiac amyloidosis, age ≥ 75 years	Up to 10% with carfilzomib + dexamethasome 4% with bortezomib + dexamethasone	Dimopoulos Lancet Oncol. 2016 [79] Das et al. Hematol. Oncol. 2022 [80] Georgiopoulos et al. JACC CardioOncology 2023 [81] Waxman et al. JAMA Oncol. 2018 [82] Russel et al. Blood 2015 [83]
**BRAF inhibitors** Vemurafenib Dabrafeniband Encorafenib **MEK inhibitors** Trametinib Cobimetinib Binimetinib Selumetinib	BRAF-mutated melanoma	Cardiomyocyte RAF-MEK extracellular signal-regulated kinases (ERKs) pathway modulation. Impact on myocyte hypertrophy, cardiac remodeling, and myocardial cell death	Previous HF, CTR-CVT, previous anthracyclines exposure, LVEF < 50%, cardiac amyloidosis, age ≥ 75 years	2% to 12% incidence of HF	Glen et al. JACC CardioOncology 2022 [84] Mincu et al. JAMA Netw. Open 2019 [85] Bronte et al. Pharmacol. Ther. 2018 [86]
***EGFR inhibitors*** Osimertinib	Non-small-cell lung cancer	Irreversible EGFR-TKI, leading to a cross-inhibition of HER2 as a potential mechanism of cardiotoxicity	Pre-existing hypertension and older age	5.5% incidence of HF	Chitturi et al. Curr. Oncol. Rep. 2022 [88] Anand et al. JACC CardioOncology 2019 [89]
**Immune checkpoint inhibitors** CTLA-4 blockers: Ipilimumab Tremelimumab PD-1 blockers: Nivolumab Cemiplimab Pembrolizumab PD-L1 blockers Atezolizumab Avelumab Durvalumab	Several kinds of cancers	Overactivation of T cells in the myocardium leading to immune-related adverse events	Dual ICI therapy, combination ICI therapy with other cardiotoxic therapies, and patients with ICI-related non-CV events or prior CTRCD or CVD	8% incidence of HF	Postow et al. J. Clin. Oncol. 2015 [90] Lyon et al. Lancet Oncol. 2018 [91] Rubio-Infante Eur. J. Heart Fail. 2021 [92] Dolladille et al. Eur. Heart J. 2021 [93] Escudier et al. Circulation 2017 [94] D’Souza et al. Eur. Heart J. 2021 [95]

ABL, Abelson oncogene; BCR, breakpoint cluster region; BRAF, B-type rapidly accelerated fibrosarcoma; CTLA, cytotoxic T lymphocyte-associated antigen-4; CTR-CVT, chemotherapy-related cardiovascular toxicity; EGFR, epidermal growth factor receptor; LV, left ventricle; LVEF, left ventricular ejection fraction; HER 2, human epidermal growth factor receptor 2; HF, heart failure; MEK, mitogen-activated extracellular signal-regulated kinase; PD-1, programmed death-1; PDL-1, programmed death-ligand 1; PE, pulmonary embolism; PI, proteasome inhibitors; Ref, reference; SERCA, sarcoplasmic reticulum calcium handling; TKI, tyrosine kinase inhibitors; VEGF, vascular endothelial growth factor; VTE, venous thromboembolism.

**Table 3:** Summary of the main cited clinical trials focusing on the prevention of heart failure in cancer patients (reviews and meta-analyses are excluded from this table).

Name of the Trial (If Any) Author, Journal, Year (Ref)	Trial Design	Population	Endpoints	Results
OVERCOME trial Bosch et al., JACC, 2013 [98]	Randomized, controlled study	90 patients with malignant hemopathies needing treatment without LVSD	Absolute change in LVEF in patients receiving carvedilol + enalapril vs. placebo	Candesartan, but not metoprolol, provides protection against early decline in LVEF
PRADA trial Gulati et al., European Heart Journal, 2016 [99]	Randomized placebo-controlled, double-blind trial	130 women with breast cancer receiving anthracyclines with or without trastuzumab assigned to candesartan, metoprolol, or matching placebo	Change in LVEF via cardiac magnetic resonance imaging	Candesartan, but not metoprolol, provides protection against early decline in LVEF
Guglin et al. JACC 2019 [100]	Double-blind, multicenter, placebo-controlled trial	468 women with breast cancer receiving trastuzumab with or without anthracyclines, randomized to receive lisinopril, carvedilol, or placebo	Average change in mean LVEF over time and distribution of cardiotoxicity for each group	Cardiotoxicity-free survival was longer on both carvedilol (hazard ratio: 0.49; 95% confidence interval: 0.27 to 0.89; p ¼ 0.009) and lisinopril (hazard ratio: 0.53; 95% confidence interval: 0.30 to 0.94; p ¼ 0.015) than on placebo
CARE trial Henriksen et al. Circulation 2023 [103]	Multicenter, prospective, randomized, open-label, blinded end-point trial	175 high-risk patients with breast cancer and non-Hodgkin lymphoma receiving anthracycline chemotherapy randomized to carvedilol and candesartan vs. standard care	Adjusted change in left ventricular ejection fraction at 6 months measured at CMR	The estimated mean difference in 6-month left ventricular ejection fraction between the cardioprotection and standard care groups was −0.37% (95% CI, −3.59% to 2.85%; *p* = 0.82)
MANTICORE trial Pituskin et al. J. Clin. Oncol. 2017 [106]	Double-blinded, placebo-controlled trial	94 patients with HER2-positive early breast cancer were randomly assigned to receive treatment with perindopril, bisoprolol, or placebo (1:1:1)	Change in indexed left ventricular end-diastolic volume and LVEF	Perindopril and bisoprolol prevented changes in LVEF at follow-up (β = 2.594, 95% CI: 0.495–4.693, *p* = 0.016; β = 4.560, 95% CI: 2.440–6.6800, *p <* 0.001, respectively)
Neilan et al. JAMA 2023 [113]	Double-blind multicenter randomized clinical trial	300 patients with lymphoma scheduled to receive anthracycline-based chemotherapy were randomized to atorvastatin 40 mg or placebo	Absolute LVEF decline ≥ 10% from before chemotherapy to a final value of <55% over 12 months	Prevalence of the primary endpoint: 9% (13/150) in the atorvastatin group and 22% (33/150) in the placebo group (*p* = 0.002)
Onoue et al. JACC CardioOncology 2023 [114]	Retrospective study applying propensity score matching to compare patients with or without metformin treatment	315 patients with diabetes receiving anthracyclines for breast cancer	New onset symptomatic HF occurring within 1 year of the initiation of anthracyclines	Metformin was associated with a lower incidence of HF (3.6% vs. 10.5%; *p* = 0.022; HR: 0.35; 95% CI: 0.14–0.90; *p* = 0.029) and lower mortality (HR: 0.71; 95% CI:0.50–1.00; *p* = 0.049)

CI, confidence interval; HR, hazard ration; LVSD, left ventricular systolic dysfunction; LVEF, left ventricular ejection fraction.

## Data Availability

No new data were created in this study. Data sharing is not applicable to this article.
